# Multiple Wasp Stings on a Child’s Supraglottis

**DOI:** 10.7759/cureus.31939

**Published:** 2022-11-27

**Authors:** Konstantinos Kourelis, Anastasios Goulioumis, Magdalene Tsiakou, Aikaterini Avgeri, Theodoros Kourelis

**Affiliations:** 1 Otolaryngology, Patras Children's Hospital, Patras, GRC; 2 Pediatrics, Patras University Hospital, Patras, GRC; 3 Oncology, Olympion General Clinic, Patras, GRC

**Keywords:** child and adolescent, airway emergency, toxic effect, local reaction, insect bite

## Abstract

Hymenoptera stings are notorious for producing severe anaphylaxis; localized effects (edema, erythema) are far more common, especially in children. However, even an innocent focused lesion may be life-threatening when the sting is directed to the airway. We present the case of a child enduring consecutive wasp stings on the supraglottis.

## Introduction

Hymenoptera stings constitute the second most frequent cause of anaphylactic reactions, ranking only behind drugs, and are justifiably feared by the public [1]. Of note, children manifest anaphylaxis due to insect stings at a much lower rate (0.3-1.0% of all pediatric systemic allergic reactions) than adults (3-34%). Less intense, localized responses are usually self-limiting and do not require particular attention [2]. Even so, depending on the anatomical location of the sting, patient’s condition may worsen considerably. Hereby we present an unusual case of a child suffering from repeated wasp stings on the supraglottic larynx.

## Case presentation

**Figure 1:**
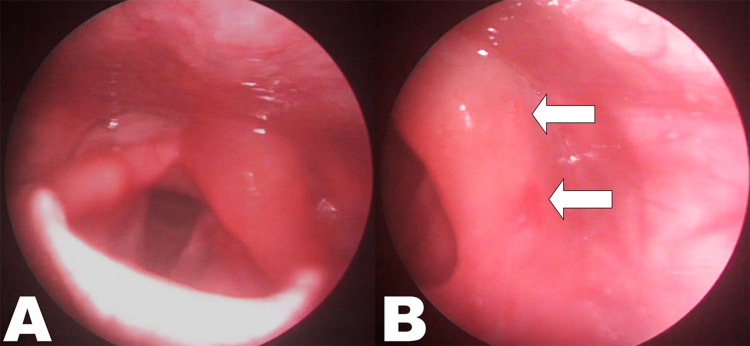
Laryngoscopic images of the child upon presentation (A) Pronounced flaccid edema of the entire left aryepiglottic fold; (B) Closer view of its lateral surface exposes two discrete sting marks (arrows)

A 13-year-old male presented at the emergency department complaining of acute mild dyspnea and burning sensation in the throat. The patient reported riding his bicycle along the countryside when an insect flew into his mouth and immediately he felt several distinct pricking sensations in his throat. Progressive throat tightness and mild dyspnea ensued in the next few minutes. The patient denied having dysphagia, cough, dizziness, or itching. Owing to his agitation at that time, the adolescent did not notice whether the insect finally escaped out through the mouth or not. On physical exam, apart from a mild throat irritation, the boy did not appear to be in distress, and his voice was not hoarse. Vital signs were within normal limits, except for mild elevation of his blood pressure (140/85 mmHg). Examination of the oral cavity and oropharynx was unremarkable for asymmetry and signs of injury or inflammation. Flexible laryngoscopy revealed marked edema and erythema of the left aryepiglottic fold (Figure 1A) and on closer inspection, two sting marks along its lateral surface (Figure 1B).

Nonetheless, glottic entrance was patent and true vocal cord mobility did not seem impaired. Endoscopic scrutiny of the upper airway for an entire insect or parts of it was negative. Complete blood count, biochemistry, and arterial blood gases, as well as an anteroposterior X-ray of the chest appeared normal. After receiving oral analgesics, intravenous dimetindene maleate, and methylprednisolone, the child was admitted for observation and respiratory monitoring. His course was uneventful with gradual relief of dyspnea, and on repeat laryngoscopy 12 hours later, complete resolution of the edema was noted. The patient was discharged on the following day with a prescription of antihistamines and methylprednisolone. On his follow-up visit to the clinic five days later, he remained symptom and sign free.

## Discussion

Insect stings account for 400,000 yearly visits to the emergency departments in USA. Of these, 40 to 100 incidents have a fatal outcome, as a result of overwhelming systemic effects, mainly in the form of fulminant anaphylaxis, triggered by the massive  Immunoglobulin E-mediated release of histamine and other inflammatory agents as a hypersensitive response to the allergenic ingredients of the venom, mostly enzymes serving as peptide antigens [1]. Children typically present with localized reactions, in the form of pain, edema, and erythema, which may peak at 24-48 hours, but nevertheless carry a minimal risk. Local responses are not allergic in nature, though histamine-driven, and are precipitated by the toxic ingredients of the venom (e.g. acetylcholine, kinins, and vasoactive amine substances). On that account, their full effect depends on the whole amount of venom, that is, the total number of stings suffered by the victim [1,3]. In the present case, at least two adjacent sting marks were recognized, indicating an increased toxic burden. Multiple stings are the hallmark of the attack by a wasp as opposed to a bee, since the latter possesses a barbed stinger that anchors into the tissue of the organism being attacked and, therefore, a second penetration is no longer possible [4]. Wasps are frequently encountered in the Greek territory, and children are heavily exposed to them due to their enthusiasm for outdoor activities.

Hymenoptera stings along the airway mucosa are extremely rare and only few cases have been described in adults, target organ being either the soft palate [5,6] or the supraglottic larynx [7-10]. All of the reported victims manifested localized reactions, and other than minor airway compromise, did not demonstrate significant complications. Only one of them was stung twice, specifically on the uvula [5]. In three cases, part of the bee remained lodged onto the epithelial lining [5,8,9]. The pediatric patient presented here sustained two consecutive stings of the supraglottic larynx, and the cumulative injected amount of venom placed him at risk of severe obstructive edema.

Insect stings of the mucosa, centered in the pharyngeal or laryngeal regions, constitute true airway emergencies, and children may be more vulnerable to the subsequent obstructive edema due to their narrower lumen. Therefore, it is imperative that patients be closely monitored for respiratory distress, receive oxygen upon presentation, and be treated urgently with intravenous antihistamines as quick onset agents. Readiness for endotracheal intubation is paramount [8]. In addition to the airway compromise, systemic reactions of twofold etiology should be anticipated. First, venom-mediated toxic effects could rarely escalate to difficult situations such as hemolysis, rhabdomyolysis, renal failure, and myocardial or cerebral infarction. Second, the more familiar, immune-mediated responses to the allergenic substances of the sting poison might be as severe as generalized hives, hypotension, bronchospasm, up to allergic shock [1,2]. Obviously, critically complicated cases call for multimodal management by a team of specialists.

## Conclusions

Emergency department physicians and otolaryngologists managing such unique cases of internal insect stings need to be aware of a few points that merit attention. First, localized venom-induced edema, although benign for the most part, is anatomy-sensitive and may prove lethal when afflicting the airway. Second, an insect flying by misfortune its way into the upper respiratory tract eventually becomes entrapped in a tight space, and is thus likely to direct a number of stings. More than that, facing great difficulty to exit the body via the oral route, it could be either inhaled, ingested, or stuck onto the upper airway walls. In any case, besides the consequences of toxic tissue damage, there is an additional risk of a retained foreign body. Hence, treating physicians should consistently scrutinize the fate of the attacking insect.
